# Attentional and working memory performance following alcohol and energy drink: A randomised, double-blind, placebo-controlled, factorial design laboratory study

**DOI:** 10.1371/journal.pone.0209239

**Published:** 2019-01-09

**Authors:** Sarah Benson, Brian Tiplady, Andrew Scholey

**Affiliations:** 1 Centre for Human Psychopharmacology, Swinburne University of Technology, Melbourne, VIC, Australia; 2 Mobile Cognition, Edinburgh, United Kingdom; Chiba Daigaku, JAPAN

## Abstract

Alcohol mixed with energy drinks (AMED) studies have typically not shown antagonism of acute alcohol effects by energy drink (ED), particularly over relatively short time frames. This study investigated the effects of alcohol, ED, and AMED on attentional and working memory processes over a 3 h period. Twenty-four young adults took part in a randomised, double-blind, placebo-controlled, factorial, 4-arm study. They were administered 0.6g/kg alcohol and 250 ml ED (containing 80 mg caffeine), and matching placebos alone and in combination. A battery of attentional and working memory measures was completed at baseline then 45, 90 and 180 min post-treatment. Alcohol produced a characteristic shift in speed/accuracy trade-off, having little effect on reaction times while increasing errors on all attentional measures (4-choice Reaction Time, Number Pairs and Visual Search), as well as a composite Attentional error score and one working memory task (Serial Sevens). ED alone improved two working memory measures (Memory Scanning accuracy and Digit–Symbol reaction times) and improved speed of responding on a composite Working Memory score. There was no consistent pattern of AMED vs. alcohol effects; AMED produced more errors than alcohol alone on one attentional measure (Visual Search errors) at 45 min only whereas AMED resulted in fewer errors on the Serial Sevens task at 90 min and better Digit-Symbol accuracy and reaction time at 45 min. Alcohol consumption increases error rate across several attentional and working memory processes. Mutual antagonism between alcohol and ED showed no consistent pattern and likely reflects a complex interaction between caffeine and alcohol levels, phase of the blood alcohol limb, task domain and cognitive load.

## Introduction

The term ‘Energy Drink’ (ED) is used to describe caffeinated beverages which are consumed on the premise that they improve alertness and physical and/or mental performance. They typically contain 80 mg of caffeine (though the levels can be much higher depending on brand and country) and other potentially psychopharmacologically active agents including glucose, taurine, glucuronolactone and B complex vitamins. The most common demographic of ED consumption are young adults [1, 2] who use them when studying, to increase energy levels, to overcome fatigue or to mix with alcohol [3].

In the context of mixing with alcohol, it has been suggested that EDs may compensate for the subjective and/or functional impairment caused by alcohol intoxication. This has triggered growing research attention over the past decade (e.g. [4–8]).Reversal of adrenergic antagonist clonidine impairments by caffeine have been observed [9]. Thus, superficially, it seems plausible that consumption of ED, containing the Central Nervous System (CNS) stimulant caffeine, might counteract the intoxicating effect of alcohol, a CNS depressant. Interestingly, self-rated alcohol intoxication has been consistently demonstrated to remain unaffected by co-administration of caffeine [6, 10–13]. On the other hand, the picture for reversal of alcohol-associated cognitive impairment by caffeine is less clear (e.g. [14–17]).

ED consumption enhances cognitive performance, particularly in tasks measuring attention and memory [14, 18–22]. This effect is usually attributed to ED’s caffeine content [4, 23–25], with the literature typically reporting that caffeine improves reaction time, attention and memory [26–32], especially in fatigued individuals. Caffeine acts as an adenosine receptor antagonist, increasing central catecholamine release resulting in increased arousal and improved cognitive functioning [9, 11, 33].

Alcohol intoxication impairs many aspects of information processing and cognition [15, 34, 35], including attention and working memory processes. It has been theorised that alcohol consumption leads to an idiosyncratic shift in speed/accuracy trade-off (SATO) often resulting in a decrease in accuracy while leaving reaction time relatively unaffected [16, 17, 36, 37]. This SATO effect may be mediated by alcohol ‘uncalibrating’ participants [38], such that they underestimate their ongoing error rate and hence do not compensate by slowing responses to decrease errors.

The mechanisms of action underlying the effects of alcohol involve various neurotransmitter systems. However the effects are complex, with different neuroanatomical loci being differentially affected by alcohol in a dose-dependent manner [39]. The primary central pharmacological effect of alcohol is to increase the functional activation of the inhibitory neurotransmitter, γ-Aminobutyric acid (GABA), leading to increased central inhibition and CNS depression [34].

Several studies have examined the cognitive effects of co-consuming alcohol and caffeine, including in the form of ED. Mackay et al [16] found that caffeine antagonised the effects of alcohol on a digit symbol substitution task but not fixed or random sequence reaction time tasks. Attwood et al. [24] reported that caffeine did not antagonise alcohol impairment in a simple reaction time or Stroop task but did reduce alcohol-related errors during a more effortful stop-signal task. In a study investigating ED and alcohol, Marczinski et al [8] reported that ED did not antagonise alcohol intoxication on any task, including a Psychological Refractory Period task, simple auditory discrimination and a pegboard task. Peacock et al [40] compared alcohol alone with 500 or 750 ml ED (160 and 240 mg caffeine respectively) over a 195 min post-drink period. Performance on a range of cognitive tasks was measured at peak target 0.08% BAC (Blood Alcohol Concentration) as well as during the rising and falling limb of the blood alcohol curve. Most effects occurred during the falling limb where both alcohol mixed with energy drink (AMED) conditions antagonised the impairing effects of alcohol on a Digit-Symbol task and a tracking task. Stop Signal task performance was antagonised by the higher caffeine drink only during the peak target 0.08% BAC. While providing some insights into the differential effects of caffeine on alcohol impairment, the study did not include an ED only arm which is important as it has been suggested that only caffeine-sensitive tasks will be susceptible to alcohol-caffeine antagonism [41]. Thus an ED non-alcohol arm can help to determine of whether apparent ED antagonism of alcohol is simply caffeine enhancement of performance independent of alcohol.

The current study aimed to investigate the effects of alcohol and ED (individually and combined) on cognitive performance and to determine whether alcohol and ED co-consumption results in antagonism of alcohol-associated impairment. Studies have investigated this relationship using caffeine but few have used complete energy drinks, hence any interaction effects of the ED ingredients have not been well researched. Many of these studies have used soft drinks as a control rather than a sensorily similar placebo [4, 8, 10] which, given the distinctive taste of many EDs, may have confounded results. With the exception of Peacock et al (2015), previous studies into the interactions of ED components with alcohol have typically not examined interactions for longer than 120 min [42]. Given that the half-life of caffeine is 4–5 h and alcohol intoxication follows a biphasic course over several hours, the present study included testing over three hours following consumption.

In real-life settings individuals tent to drink alcohol to an intoxication end-point (reflected by administering different quantities and concentrations of alcohol in different beverages such as spirits, wine and beer). Regarding caffeine, however, they typically administer fixed amounts–especially when this is in the form of carbonated caffeinated beverages such as energy drinks. This is reflected in the literature where typically alcohol is administered on a dose/weight basis but fixed levels of caffeine are administered [11, 12, 40, 43–45]. In order to allow comparison with extant literature in this field, the same approach was adopted here. In this case using a single typical energy drink (80 mg caffeine) and alcohol aimed to produce BACs at the threshold of the drink-driving limit (0.05%).

Based on previous literature, we hypothesised firstly that any antagonism of alcohol impairment by caffeine may be more evident during the falling arm of the blood alcohol limb and secondly that the same tasks which were enhanced by ED alone would have any alcohol impairment reversed by combination with ED.

Additionally, this study aimed to examine the presence of any treatment-related SATO and to investigate whether it would be differentially affected by co-consumption of AMED compared with alcohol alone. Finally, given that the few studies which have found antagonism of alcohol impairment by caffeine and/or ED [16, 24] have tended to be on relatively more effortful tasks, we were interested in the interactions of any effect with cognitive load.

## Method

The study was approved by the Swinburne University Human Research Ethics Committee (SUHREC). Approval number 2011/139.

### Design

The present study utilised a factorial, double-blind, placebo-controlled, crossover design and was part of an evaluation of alcohol and energy drinks on mood and cognitive performance. The mood data have been published elsewhere [12]. The study was registered on the Australian New Zealand Clinical Trials Registry (ACTRN12612000295842).

### Participants

Twenty-four participants (12 females) with a mean age of 22.83 years (range 18–40) and mean weight of 71.38 kg (range 50–100) were recruited via social media, flyers and word-of mouth.

All participants were healthy, not taking any prescribed medications (except the contraceptive pill in 50% of females). They were regular (i.e. daily) caffeine consumers as determined by a short verbal questionnaire, and regular alcohol consumers. Exclusion criteria included current or past alcohol abuse, or current or past psychiatric disorders.

### Treatments

Alcohol, in the form of Vodka (40% alcohol by volume) was administered at 0.6g/kg body weight in the aim of achieving a peak BAC of 0.05%. In the placebo alcohol conditions, vodka was wiped around the rim of the glass and water was added in replacement of the vodka to avoid any sensory discrepancies. The ED treatment was a standard 250 ml can of Red Bull (Salzburg, Austria) containing 80 mg caffeine. In the placebo energy drink condition, a Red Bull minus all active ingredients but identical in flavour, colour and smell was used. Participants were randomly assigned to a condition sequence so that each participant received ALC (0.6g/kg alcohol, 250 ml placebo energy drink), ED (0.6g/kg water, 250 ml energy drink), AMED (0.6g/kg alcohol, 250 ml energy drink) and PLA (0.6g/kg water, 250 ml placebo energy drink) over four testing sessions.

### Blood alcohol concentration

Breathalyser readings were taken using a Lion Alcolmeter SD400PA by an uninvolved third party who concealed the readings from the participant and researcher until the conclusion of the study.

### Computerised test battery

A computerised test battery was used to evaluate treatment effects on attention (Four-Choice Reaction Time, Number Pairs, Visual Search) and working memory (Memory Scanning, Serial Sevens, Digit-Symbol Matching, Visuospatial Working Memory).

Tasks were completed using an Acer Iconia Tablet. All responses were made by tapping on the screen and were automatically recorded. The tasks were selected for their previously demonstrated sensitivity to alcohol, energy drink or caffeine [15–17, 46] and were presented as detailed below.

#### Four-choice reaction time (FCRT)

A 2 x 2 stimulus array of circles at the top-end of the screen corresponded to a response array of four squares at the lower-end of the screen. The circles lit up one at a time and the participant had to tap the corresponding square. The task was scored for reaction time and number of errors made.

#### Memory Scanning

Participants were presented with a series of 5-digit arrays. After each set of digits, they were presented with a series of digits one at a time and had to indicate whether or not each was in the original set. Reaction times and number of errors were recorded.

#### Number Pairs

A series of five-digit arrays were presented on the screen. For each array, the participant had to respond whether the second and fourth digits were the same. Yes/No responses were recorded and the task was scored for reaction time and number of errors made.

#### Serial Sevens

Participants were presented with a three-digit number between 800 and 999. When they indicated they were ready another number appeared and the participant had to indicate whether or not the new number was 7 lower than the previous. The operation continued for 2 min. The number of responses and errors made were recorded.

#### Digit-Symbol Matching

A key was presented on the screen displaying the digits one through to nine with a unique symbol corresponding to each. Below this was a series of digit-symbol pairs appearing sequentially and the participant had to indicate whether it matched the key by selecting ‘yes’ or ‘no’. Reaction time and errors made were recorded.

#### Visual Search

A 6 x 6 array of letters in different orientations appeared on the screen. For each screen the participant must locate the target, which was an **L** shape in any orientation, and tapped on it as quickly as possible. Non-target shapes were **T**. Reaction time and errors were recorded.

#### Visuospatial Working Memory (VSWM)

A series of two arrays of four pictures were presented on the screen. Each array was displayed for five seconds with a delay of two seconds between each array. Two of the four pictures in the first array were the same. The second array contained four pictures, two of which were the same as the first and one that was in the same position while the second was in a different position. A third array appeared on the screen containing eight pictures and participants were asked one of the following: “Tap on the picture that was shown in both displays IN DIFFERENT POSITIONS”, “Tap on the picture that was not shown IN EITHER DISPLAY”, “Tap on the picture that was shown in both displays IN THE SAME POSITION” or “Tap on the picture that was shown twice IN THE SAME DISPLAY”. The task was scored for errors and reaction time.

### Procedure

Each participant attended an initial screening session plus four testing sessions that were conducted with a minimum 48-hour washout period. Treatment order was fully counterbalanced and determined by random allocation to a sequence order according to a Latin Square design. During the screening session, participants provided written informed consent before undergoing an assessment of their eligibility. Participants were told to refrain from any alcohol and caffeine twelve hours prior to testing and should eat a similar meal one hour prior to each of the four testing sessions. Participants then completed two shortened versions of the cognitive testing battery.

At the beginning of each testing session, participant’s caffeine, food and medication intake was assessed. Participants then underwent baseline testing involving BAC testing to ensure a reading of 0.00% and then completed the cognitive testing battery. Drinks were administered and participants were given 10 minutes consumption time. BAC’s were taken again at 45 and 210 minutes post drink. The cognitive battery was completed at 45, 90 and 180 minutes post drink. Participants remained at the Swinburne University Centre for Human Psychopharmacology for the duration of the sessions and were prohibited from eating or drinking anything other than water.

### Statistical analyses

All analyses were performed using IBM SPSS Version 21. To evaluate the possibility that differences in baseline scores may influence post-treatment measures, baseline scores under each condition were analysed using a series of one-way, repeated measures ANOVAs resulting in no significant differences. Change-from-baseline scores were computed for each outcome. Primary analyses were undertaken using a 2 (Alcohol; alcohol, placebo alcohol) x 2 (Energy Drink; energy drink, placebo energy drink) x 3 (Time; 45 min, 90 min and 180 min) repeated measures ANOVA. Significant main effects and interactions were further investigated using paired samples *t*-tests comparing treatments at each time point. All testing was two-tailed and comparisons were planned prior to testing. No correction was made for multiple testing as we wished to ensure that any potentially harmful alcohol/ED interactions were captured. In order to examine whether relationships held with reduced possibility of Type I error, the same statistical analyses were applied to aggregate cognitive scores derived from the attention (FCRT, Number Pairs, Visual Search) and working memory (Memory Scanning, Serial Sevens, Digit-Symbol VSWM) tasks. These produced four aggregate (mean) measures; Attention errors, Attention reaction time, Working Memory errors and Working memory reaction time.

Effect sizes (Cohen’s d) were calculated for the significant *t*-tests using ‘Equation 8’ [47]. To control for dependence in the data ‘Equation 8’ was not applied to the effect size calculation for Digit-Symbol Matching at 180 min as the mean and standard deviation in the placebo condition were 0.00 and hence could not be correlated.

## Results

There were no significant differences in any baseline measure.

### BAC

BACs from this study have been reported previously [12]. All participants recorded a baseline BAC reading of 0.00% across all conditions and, as expected, there were significant main effects of time [*F*(1,23) = 482.07, *p* < .001] and alcohol [*F*(1,23) = 209.00, *p* < .001] and a significant alcohol x time interaction [*F*(1,23) = 482.07, *p* < .001]. At 45 min, there were significant differences between the ALC and AMED treatments compared to PLA [ALC: *t*(23) = 15.33, *p* < .001, *d* = 4.43; AMED: *t*(23) = 21.11, *p* < .001, *d* = 6.07] and ED [ALC: *t*(23) = 15.33, *p* < .001, *d* = 4.43; AMED: *t*(23) = 21.11, *p* < .001, *d* = 6.07]. Similarly at 210 min, significant differences were found between the ALC and AMED treatments compared to PLA [ALC: *t*(23) = 7.23, *p* < .001, *d* = 2.09; AMED: *t*(23) = 6.62, *p* < .001, *d* = 1.91] and ED [ALC: *t*(23) = 7.23, *p* < .001, *d* = 2.09; AMED: *t*(23) = 6.62, *p* < .001, *d* = 1.91]. There were no differences between the ALC and AMED treatments at 45 min [*t*(23) = 1.44, *p* = 0.163] or 210 min [*t*(23) = 1.24, *p* = 0.227].

### Cognitive battery

All data were successfully recorded with the exception of reaction time in the FCRT task due to software failure. There were main effects of time on a number of outcomes, however, for the purpose of brevity, we will restrict ourselves to reporting statistics for main effects of treatment and treatment x time interactions and differences between treatments at each time point only.

### Individual tasks scores

#### Attentional measures

**Fig 1:**
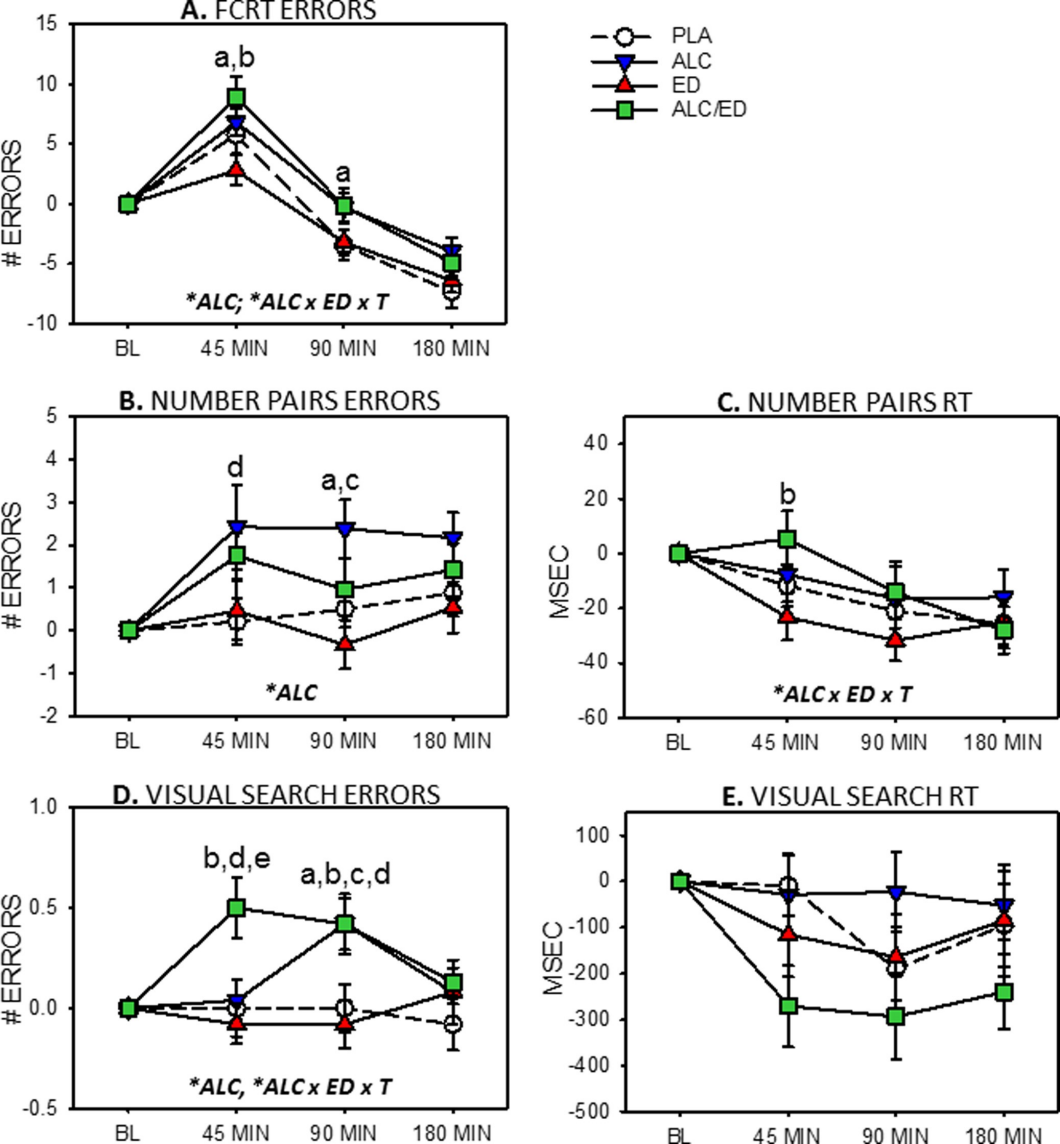
Attentional effects of alcohol (ALC), energy drink (ED) and alcohol mixed with energy drink (AMED). Graphs depict means (with SEM) change-from-baseline error (left hand column) and reaction time (RT, right hand column). Capitalised, italicised letters in panels depict significant main effects of alcohol (ALC) and alcohol x energy drink x time interactions (ALC x ED x T). Lower case letters signify between-treatment significance at each time point (a, ALC vs. ED; b, AMED vs. ED; c, ALC vs. PLA; d, AMED vs. PLA; e, AMED vs. ALC).

Errors and reaction times for attentional tasks are presented in Fig 1.

#### Four-choice Reaction Time (FCRT)

For FCRT errors (the primary outcome), there was a main effect of alcohol [*F*(1,23) = 7.92, *p* = .010] with more errors in the alcohol conditions. There was also a significant alcohol x ED x time interaction [*F*(2,46) = 3.26, *p* = .048]. Significantly more errors made in the ALC compared to the ED treatment at 45 [*t*(23) = 2.97, *p* = .007, *d* = 0.607] and 90 min [*t*(23) = 2.39, *p* = .025, *d* = 0.490]. At 45 min, significantly more errors were made in the AMED compared to the ED treatment [*t*(23) = 2.71, *p* = .013, *d* = 0.558], see Fig 1A.

#### Number Pairs

For Number Pairs errors, there was a significant main effect of alcohol [*F*(1,23) = 7.02, *p* = .014] with alcohol being associated with more errors. There was a trend for more errors to be made in the ALC compared to the PLA treatment at 45 min [*t*(23) = 2.05, *p* = .051, d = 0.445] and significantly more errors in the ALC compared to the PLA treatment at 90 min [*t*(23) = 2.54, *p* = .018, *d* = 0.539]. Also at 45 min, significantly more errors were made in the AMED compared to the PLA treatment [*t*(23) = 2.44, *p* = .023, *d* = 0.498]. Finally, significantly more errors were made in the ALC compared to the ED treatment at 90 min [*t*(23) = 2.82, *p* = .010, *d* = 0.578] and a trend for the same relationship at 180 min [*t*(23) = 2.07, *p* = .050, d = 0.424], see Fig 1B.

Regarding Number Pairs reaction time, there was a significant alcohol x ED x time interaction [*F*(2,46) = 4.19, *p* = .021]. Further analyses revealed that the only significant group difference occurred at 45 min with faster reaction time in the ED compared to the AMED treatment [*t*(23) = 2.45, *p* = .034, *d* = 0.464], see Fig 1C.

#### Visual Search

For Visual Search errors there was a significant main effect of alcohol [*F*(1,23) = 8.52, *p* = .008] with more errors in the alcohol conditions. There was also a significant alcohol x ED x time interaction [F(2,46) = 3.88, *p* = .028] and a trend for an alcohol x time interaction [*F*(2,46) = 2.96, *p* = .062]. At 45 min, significantly more errors were made in the AMED compared to the ALC [*t*(23) = 3.11, *p* = .005, *d* = 0.667], PLA [*t*(23) = 2.15, *p* = .043, *d* = 0.437],and the ED [*t*(23) = 3.08, *p* = .005, *d* = 0.632] treatments. At 90 min, significantly more errors were made in the ALC compared to the PLA [*t*(23) = 2.63, *p* = .015, *d* = 0.545] and ED treatments [*t*(23) = 2.40, *p* = .025, *d* = 0.491]. Also at 90 min, significantly more errors were made in the AMED compared to the PLA [*t*(23) = 2.20, *p* = .038, *d* = 0.452] and ED treatments [*t*(23) = 3.14, *p* = .005, *d* = 0.642], see Fig 1C.

There were no significant effects on Visual Search reaction time (Fig 1D).

### Working memory measures

**Fig 2:**
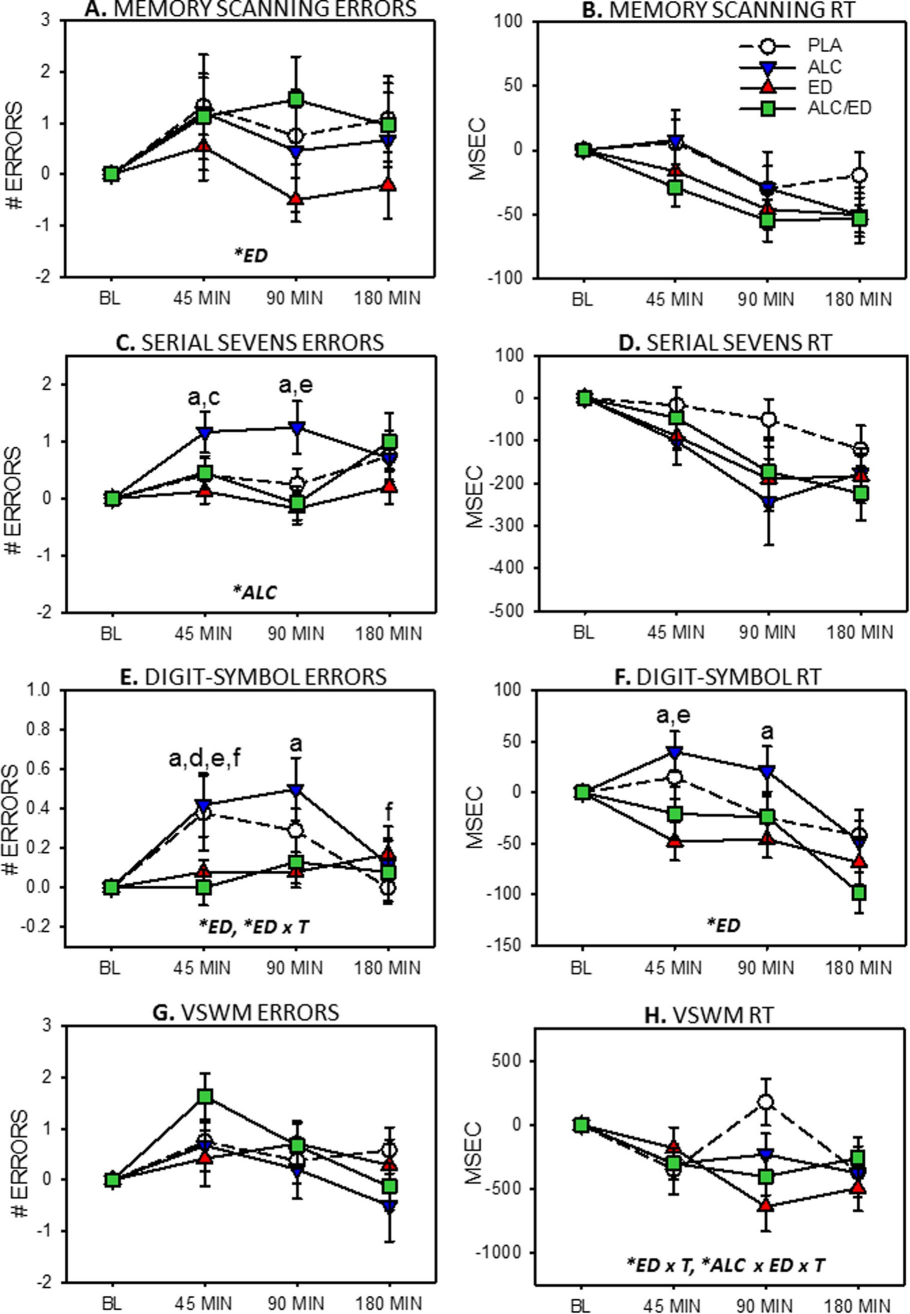
Working memory effects of alcohol (ALC), energy drink (ED) and alcohol mixed with energy drink (AMED. Graphs depict means (with SEM) change-from-baseline error (left hand column) and reaction time (RT, right hand column). VSWM = Visuospatial working memory. Capitalised, italicised letters in panels depict significant main effects of energy drink (*ED*), alcohol (ALC), energy drink x time (*ED x T*) and alcohol x energy drink x time interactions (*ALC x ED x T*). Lower case letters signify between-treatment significance at each time point (a, ALC vs. ED; c, ALC vs. PLA; d, AMED vs. PLA; e, AMED vs. ALC; f, ED vs. PLA).

Errors and reaction times for working memory tasks respectively are presented Fig 2.

#### Memory Scanning

There was a main effect of ED [*F*(1,23) = 5.42, *p* = .029] on Memory Scanning accuracy with fewer errors in the ED conditions (see Fig 2A). However, further analysis found that there were no significant differences at any time point nor any effect on Memory Scanning reaction time (Fig 2B).

#### Serial Sevens

For Serial Sevens errors there was a significant main effect of alcohol, [*F*(1,23) = 4.62, *p* = .042] with alcohol being associated with a higher error rate. At 45 min, more errors were made in the ALC treatment compared to the PLA [*t*(23) = 2.10, *p* = .047, *d* = 0.435] and ED [*t*(23) = 2.44, *p* = .023, *d* = 0.511 ] treatments. At 90 min, more errors were made in the ALC compared to the AMED [*t*(23) = 2.48, *p* = .021, *d* = 0.517], and ED [*t*(23) = 2.28, *p* = .032, *d* = 0.609] treatments, see Fig 2C. There were no significant effects on Serial Sevens RT (Fig 2D).

#### Digit-Symbol Matching

For Digit-Symbol Matching errors there was a significant main effect of ED [*F*(1,23) = 5.97, *p* = .023] where ED was associated with fewer errors. There was also a significant ED x time interaction [*F*(2,46) = 5.31, *p* = .008]. At 45 min, fewer errors were made in the ED compared to the ALC [*t*(23) = 2.15, *p* = 0.43, *d* = 0.519] and PLA [*t*(23) = 2.60, *p* = .016, *d* = 0.606] treatments. Additionally at 45 min, fewer errors were made in the AMED compared to the ALC [*t*(23) = 2.46, *p* = .022, *d* = 0.535] and PLA [*t*(23) = 2.84, *p* = .009, *d* = 0.598] treatments. At 90 min, again, fewer errors were made in the ED compared to the ALC [*t*(23) = 2.46, *p* = .022, *d* = 0.791]. Finally, at 180 min, more errors were made in the ED compared to the PLA treatment [*t*(23) = 2.15, *p* = .043, *d* = 0.438], see Fig 2E.

Regarding Digit-Symbol reaction times, there was a significant main effect of ED [*F*(1,23) = 4.82, *p* = .038], again with faster performance in the ED conditions. There was also a trend towards an alcohol x time interaction [*F*(2,46) = 3.08, *p* = .055]. At 45 min, responses were significantly quicker in the ED compared to ALC treatment [*t*(23) = 2.75, *p* = .001, *d* = 0.561] and in the AMED compared to the ALC treatment [*t*(23) = 2.27, *p* = .033, *d* = 0.472]. At 90 min, there was a trend for quicker responses in the ED compared to ALC treatment [*t*(23) = 2.03, *p* = .054, *d* = 0.181], see Fig 2F.

#### Visuospatial Working Memory

There were no effects on Visuospatial Working Memory errors (Fig 2G). For Visuospatial Working Memory reaction time, there was a significant ED x time interaction, [*F*(2,46) = 4.42, *p* = .018] and a significant alcohol x ED x time interaction [F(2,46) = 3.24, *p* = .048]. There were no significant differences between treatments at any time point (see Fig 2H).

### Composite scores

**Fig 3:**
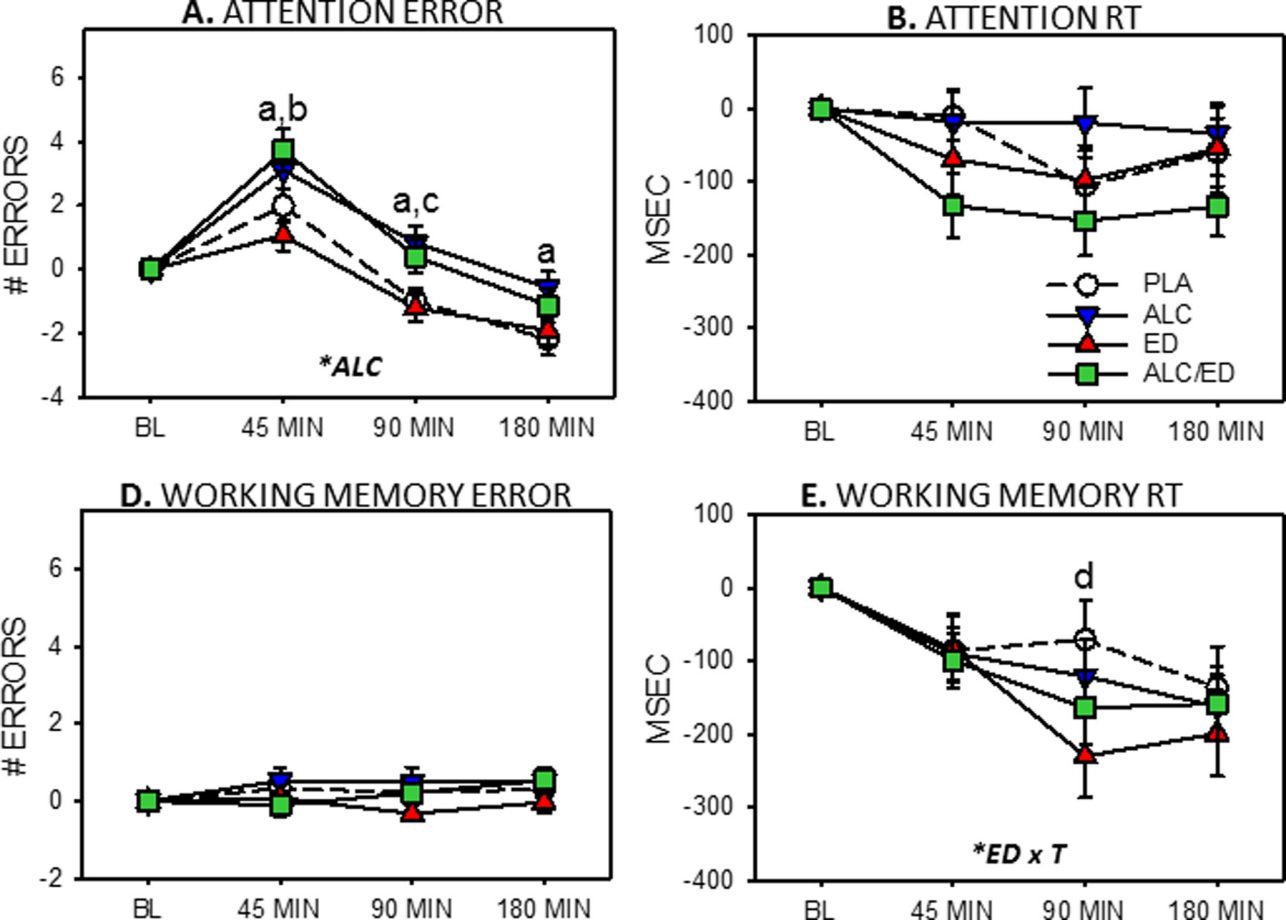
Effects of alcohol (ALC), energy drink (ED) and alcohol mixed with energy drink (AMED on composite Attention and Working Memory scores. Graphs depict means (with SEM) change-from-baseline error (left hand column) and reaction time (RT, right hand column). Capitalised, italicised letters in panels depict significant main effects of alcohol (ALC) and energy drink x time interactions (ED x T). Lower case letters signify between-treatment significance at each time point (a, ALC vs. ED; b, AMED vs. ED; c, ALC vs. PLA; d, AMED vs. PLA).

Errors and reaction times, respectively, for composite Attention and Working memory measures are presented Fig 3.

#### Attention composite

As depicted in Fig 3A, for the attention composite, there was a significant main effect of alcohol on errors [*F*(1,23) = 11.95, p = .002]. At 45 min, significantly more errors were made in the ALC compared to the ED (*t*(23) = 3.11, p = .005, d = 0.641) and AMED compared to ED (*t*(23) = 3.10, p = .005, d = 0.640) conditions. At 90 min, significantly more errors were made in the ALC compared to PLA (*t*(23) = 2.34, p = .029, d = 0.477) and ED (*t*(23) = 3.32, p = .003, d = 0.685) conditions. Also at 90 min, significantly more errors were made in the AMED compared to the ED condition (*t*(23) = 5.87, p < .001, d = 1.219). At 180 min, significantly more errors were made in the ALC compared to ED condition (*t*(23) = 2.57, p = .017, d = 0.528).

There were no significant effects on composite attention reaction time scores.

#### Working memory composite

Working memory composite showed a different pattern of results (Fig 3D and 3E). There were no significant effects on errors. For Working Memory, there was a significant ED x time interaction [F(2,46) = 4.27, *p* = .020]. At 90 min, reaction time was significantly faster in the ED compared to PLA condition [*t*(23) = 2.17, *p* = .040, *d* = 0.444]. There were no other significant differences.

## Discussion

The primary aim of this study was to investigate the effects of alcohol and ED, alone and in combination, on cognitive performance with a focus on attention and working memory. Our primary hypotheses were not supported in that differences between alcohol alone and AMED were largely evident at 45 min (and one measure at 90 min), coinciding with the rising, rather than the falling limb of the blood alcohol curve in this cohort [12]. The reasons for the difference between this and one previous report by Peacock and colleagues showing differential effects during the falling limb [40] are not obvious, but may reflect differences in levels of both alcohol and caffeine between the studies. Peacock used higher levels of alcohol (target BAC of 0.08%) and caffeine (160 and 240 mg) and, unusually, also reported that BACs were lower in the AMED compared to alcohol alone condition. This again suggests dose-specific effects—here as previously reported (Benson and Scholey, 2014) and consistent with earlier findings [8, 10, 11], BACs post administration of AMED were indistinguishable from those following alcohol.

Likewise, our hypothesis that the tasks showing greatest enhancement by the caffeinated ED would differ most between alcohol and AMED (which would be denoted by both an **e** and **f** notation at the same time point in Figs 1, 2 and 3) was not supported. This suggests that the neural mechanisms of caffeine/alcohol antagonism are somewhat more complex than those targeted by caffeine alone and are unlikely to be restricted to simple targeting of the adenosine receptor.

Turning to main effects of treatments, alcohol produced a characteristic SATO shift. Alcohol increased the composite Attention error score and errors on all attentional measures, i.e. 4-choice reaction time (the primary outcome), number pairs and visual search as well as one working memory task (Serial Sevens) while leaving reaction times relatively unaltered. Indeed, alcohol had a significant effect on one reaction time measure only–manifest as a significant interaction with ED and time on speed of responding on the Number Pairs task. The differential impairment of accuracy over speed is consistent with that from other studies of alcohol in the laboratory [16, 17, 36–38, 46] and field [17] and may result from increased confidence in performance coupled with poor “calibration” [38].

Regarding the Working Memory composite scores, there was an ED x Time interaction, whereby ED tended to produce faster scores at later testing times, which was manifest as a significant improvement over placebo at 90 min. The main effects of ED were restricted to improvement of two working memory measures—Memory Scanning accuracy and Digit–Symbol (accuracy and reaction time), the latter being consistent with previous reports [16, 40]. While this suggests that the effect may be largely driven by the caffeine in ED, it should be noted that the uncaffeinated placebo did not contain taurine or B-vitamins. It is possible that these components may have contributed to the ED vs. placebo cognitive effects. Indeed previous research has found improved working memory performance following a drink containing B vitamins and caffeine (in the form a guaraná) [48, 49]. There are few studies comparing the cognitive effects of interactions between individual ED components. Those which have done so report selective effects, with taurine partially antagonising the effects of caffeine on certain tasks only [50, 51]. This phenomenon may contribute to the limited number of tasks differentially affected by ED compared with other studies examining caffeine. Additionally, the placebo also contained glucose which enhances cognition across a range of domains [52–55]. This raises the possibility that the limited ED effects are the result of glucose in the placebo drink enhancing performance above what would be seen using an inert placebo. Another consideration is that, as is usual in caffeine studies and to maintain ecological validity, the caffeine dose was fixed rather than administered in a weight-dependent manner. Thus there may have been considerable variation in systemic and central caffeine levels. On the other hand each participant underwent every condition and consumed the same meal (at the same time where possible). Therefore, although this will have resulted in some inter-subject variability, the variability between conditions would have been minimal. Future studies might usefully measure caffeine in blood or saliva to address the relationship between caffeine levels and performance in this context.

One impetus for research in this area has been the potential functional effects of AMED compared with alcohol alone. Specifically there have been concerns that AMED may antagonise alcohol effects or exacerbate alcohol impairment leading to greater functional impairment when intoxicated. Despite statistical alcohol x ED x time interactions on three measures, there was no consistent pattern across tasks of AMED compared with alcohol. On a single attentional measure (Visual Search errors) AMED resulted in more errors than alcohol alone at 45 min only. Conversely at 90 min, accuracy was impaired in the two alcohol treatments compared to both the placebo and ED treatments. These findings are consistent with those of Marsden and Leach [56] who reported impaired performance on a similar task following 75 ml alcohol compared with 75 mg caffeine. Unlike our study, however, the authors failed to find differences between either of the treatments and placebo.

A different pattern of alcohol-caffeine interactions was found on other measures. Compared with alcohol alone, AMED resulted in fewer errors on the Serial Sevens task at 90 min and Digit-Symbol (errors and reaction time) at 45 min only. The findings on the latter measure are consistent with Mackay et al [16] using the classic, pencil-and-paper Digit-Symbol Substitution task (DSST) and somewhat larger doses of caffeine (110–120 mg) and alcohol (0.66 g/kg) than here. They found that the combination of alcohol and caffeine significantly reduced alcohol impairment in the DSST confirming the potential sensitivity of this task to caffeine-alcohol antagonism. Alcohol alone has been shown to impair Serial Sevens performance [17, 38, 57], while performance was improved by a caffeinated ED [20]. This is the first study we are aware of showing mutual antagonism of performance on this task by alcohol and caffeine. The reason for the sensitivity of these particular tasks is unknown. One possibility is that the tasks engender a similar level of cognitive load or mental effort. If one considers that baseline reaction times for responses are an index of the mental effort required for task performance, then the Serial Sevens and the Digit Symbol tasks require strikingly similar levels of effort (with mean baseline values of 1031 and 1146 msec respectively. This compares with mean baseline reaction times of 620 and 2134 msec, respectively, for the Number Pairs and Visual Search attentional tasks, and 720 and 3107 msec respectively for the Memory Scanning and Visuospatial Working Memory tasks. Thus task susceptibility to caffeine-alcohol antagonism may reflect a complex interaction between caffeine and alcohol levels, phase of the blood alcohol limb, task domain and cognitive load.

During Visuospatial Working Memory Task, there was a significant time x treatment interaction for reaction time scores, although there were no significant differences between the treatments at any time-point, making interpretation of this effect difficult.

There were several other time-specific group differences. In the FCRT task (our primary outcome), more errors were made in the ALC compared to ED treatment at 45 and 90 min post treatment. Additionally, more errors were made at 45 min in the AMED compared to the ED treatment. There we no significant differences between any of the treatments and placebo, although the changes in the ALC and AMED were in the direction of impairment while those in the ED were in the direction of improvement. These results are consistent with previous findings by Mackay et al [16] with somewhat larger doses (0.66g/kg alcohol and 110-120mg caffeine), who found that alcohol led to an increased error rate while caffeine had no effect when administered alone.

During the Number Pairs task, at 45 min more errors were made in the AMED compared to PLA treatment while at 90 minutes, more errors were made in the ALC compared to PLA. Additionally, more errors were made in the ALC compared to ED treatment at 90 and 180 min. Finally, at 45 min, reaction time was significantly quicker in the ED compared to the AMED treatment. This task has not been used previously in ED nor caffeine studies although, it has been found to demonstrate sensitivity to alcohol intoxication. In a comparison study of cognitive function in the laboratory and everyday life, Tiplady [46] found that under both conditions (BACs of 0.124% and 0.095% respectively), participants made significantly more errors in the Number Pairs task compared to when alcohol had not been consumed. Reaction times during the everyday condition, however, remained unchanged but were significantly slower in the laboratory setting. In another laboratory study an alcohol dose slightly higher than in our study (BAC of 0.077%) impaired both accuracy and reaction time [15].

No correction was made for multiple comparisons as we did not wish to minimise any potential AMED-associated impairments. In the above sections we have included all significant findings but emphasised only those which would survive adjustment for multiple comparisons. It should be noted that main effects of alcohol on all attentional measures and the Attention composite (but not on working memory) would remain following adjustment for multiple comparisons. Other attentional measures which are robust to this adjustment include the 45 min difference between alcohol and ED on FCRT errors, the AMED vs. alcohol and AMED vs. ED comparisons on Visual Search errors at 45 min, and the alcohol/placebo and AMED vs. ED comparisons on the same measure at 90 min. Regarding working memory measures, the ED x Time interaction and the 45 min AMED vs. placebo and ED vs. alcohol comparisons for Digit Symbol errors and reaction time respectively would also remain. Treatment x Time effects on the composite Working Memory score, however would not be preserved.

There were some limitations in our study. Testing sessions began either in the morning or early afternoon, although we tried to hold testing times as constant as possible within individual participants. The circadian clock may affect behavioural and physiological responses to alcohol with decreased sensitivity later in the individual’s circadian cycle [58]. A study administering young males with alcohol over different times of the day found that participants BAC levels were significantly higher in the morning compared to the evening [59]. Additionally, participants were asked to refrain from caffeine, thus reversal of caffeine withdrawal may account for certain caffeine effects (although the ED alone effects were very limited). Furthermore, while precautions were taken to ensure adequate blinding, participants may have been aware of the alcohol condition administrated and effects may have been moderated to some degree by expectancy. Lastly, we assessed mean reaction time and accuracy and did not measure particular aspects that form the response, e.g. encoding information. Previous research indicates that caffeine administration improves encoding time and that improvements in mean reaction time may be driven by decreased occurrence of attentional lapses that would otherwise result in long response times [60]. Although not the focus of the current study, future research could usefully examine the microstructure of responding during caffeine-sensitive tasks in the context of alcohol and AMED.

In conclusion, the results of this study confirm that alcohol intoxication impairs information processing according to a characteristic SATO shift reflecting reduced accuracy in the absence of effects on response times. The co-administration of ED and alcohol was found to antagonise intoxication related impairment on one task at one time point only. Further research is needed to further delineate the cognitive domains sensitive to reversal of intoxication related impairment following caffeinated alcohol and to further examine the necessity of a main effect of caffeine for antagonism to be demonstrated.
